# Sex and Rural/Urban Centre Location as Determinants of Body Image Self-Perception in Preschoolers

**DOI:** 10.3390/children9121952

**Published:** 2022-12-12

**Authors:** Jorge Rojo-Ramos, Santiago Gomez-Paniagua, José Carmelo Adsuar, Ángel Denche-Zamorano, María Mendoza-Muñoz, Sabina Barrios-Fernandez

**Affiliations:** 1 Physical Activity for Education, Performance and Health, Faculty of Sport Sciences, University of Extremadura, 10003 Cáceres, Spain; 2 BioẼrgon Research Group, Faculty of Sport Sciences, University of Extremadura, 10003 Cáceres, Spain; 3 Promoting a Healthy Society Research Group (PHeSO), Faculty of Sport Sciences, University of Extremadura, 10003 Cáceres, Spain; 4 Research Group on Physical and Health Literacy and Health-Related Quality of Life (PHYQOL), Faculty of Sport Sciences, University of Extremadura, 10003 Cáceres, Spain; 5 Departamento de Desporto e Saúde, Escola de Saúde e Desenvolvimento Humano, Universidade de Évora, 7004-516 Évora, Portugal; 6 Occupation, Participation, Sustainability and Quality of Life (Ability Research Group), Nursing and Occupational Therapy College, University of Extremadura, 10003 Cáceres, Spain

**Keywords:** body image, preschool, sex, body dissatisfaction

## Abstract

Body image and self-perception are highly related to psychological health and social well-being throughout the lifespan. Body image problems can lead to pathologies affecting the quality of life. Thus, it is essential to analyse perceived self-image from an early stage. This study aimed to assess body image and dissatisfaction in preschoolers, analyzing possible differences depending on sex (boy/girl) and school location (rural/urban). The sample consisted of 304 preschoolers from Extremadura (Spain) between three and six years of age. The Mann–Whitney U test was used to evaluate the differences in scores according to sex and centre location. The results showed significant differences in the body shape perception depending on the student’s sex, with females showing higher scores in their Body Mass Index (BMI). However, females showed greater body dissatisfaction than their male counterparts, with greater disagreement between their perceived and desired figures. Actions and programmes to promote children’s healthy body image need to be implemented with consideration for differences between the sexes.

## 1. Introduction

Positive embodiment and body appreciation are significant aspects of health and quality of life [1]. Body image describes how a person feels, thinks, and perceives their body [2], although this term is considered to be multidimensional [3]. The most commonly used body image measures are those that evaluate the person’s appreciation of his or her physical appearance [4]. Although research on body image frequently adopts a pathologizing perspective, concentrating on body dissatisfaction, the importance of considering body appreciation and positive aspects of body image has been raised in recent years [5]. Scientific literature indicates that positive body esteem is associated with self-esteem, healthy eating habits, and higher physical activity levels regardless of sex [6,7]. Likewise, body image has been found to predict health and quality of life in both boys and girls [8]. Numerous studies have revealed several aspects of positive body image, such as positive opinions, respecting and feeling grateful for one’s body, rejecting societal ideals of attractiveness, inner positivity influencing one’s outward appearance, and having a broad conception of beauty [9]. In this line, surveys with girls and women have influenced the developmental theory of embodiment [10] which considers healthy body image as a condition of body-self unification, characterized by feeling “at one” with the body. In this sense, the ages at which these ideas start to develop may be learned through studies looking at potentially detrimental attitudes about weight and body size among youth. Some research suggests that children start to become conscious of their body image and how they feel about it at the age of three [11]. Moreover, findings suggest girls aged just three are already emotionally committed to the ideal of thinness [12]. Then, youngsters between three and six already see fat negatively and favour a lean body [13]. Spiel and colleagues discovered that children between three to five years of age preferred larger figures to symbolize negative traits as opposed to good traits [14]. Thus, preschoolers must be taught healthy attitudes toward their bodies, diet, and activity to prevent body image disorders and related pathologies [15].

On the one hand, a variety of sociocultural influences are linked to body image development [16]. Thompson et al. [17] indicate that media, friends, and parents are three elements that impact the emergence of body dissatisfaction. Parents have a significant impact on how their children see their bodies by modelling attitudes and behaviours linked to beauty [18]. Hence, research has also shown a connection between children’s body dissatisfaction and peer influence, such as teasing, dialogues, or modelling [18]. Children’s body dissatisfaction has also been linked to promotion of idealized bodies in media [19]. On the other hand, biological components are also significant for children’s body image. Body mass index (BMI) has been linked to children’s body image and usage of body modification strategies [16]. Another essential factor to consider is sex, as research has shown that males and girls may have different body worries [20].

In terms of body image assessment, methods used in research with preschoolers frequently call for neither reading proficiency nor much vocal participation from participants [21]. A commonly used technique, which has been tried with kids as young as two-and-a-half years old, is to display two to three line drawings of various sizes while reading a list of words [22]. The use of a figure rating scale in an interview setting where children are presented with age- and sex-appropriate pictorial representations of a range of body sizes, from extremely thin to extremely huge, is another [23]. Children are then asked to choose the images of themselves that they believe best represent their existing appearance (current) and their ideal appearance (ideal). Also, other tools that have been regularly used are questionnaires [24].

Consequently, the objective of the present research was to evaluate body satisfaction in preschoolers in public schools from Extremadura (Spain), assessing whether there were possible differences depending on sex or the educational centre location. This will make it possible to identify the current state of body image at the most critical ages of development, and subsequently make it possible to implement educational programs and/or lines of action depending on the studied variables.

## 2. Materials and Methods

### 2.1. Participants

Participants were selected using a non-probabilistic sampling method based on convenience sampling [25]. The sample consisted of 304 preschool children (from 3 to 6 years old) from public schools in Extremadura (Spain). 

### 2.2. Instruments

A questionnaire with five sociodemographic questions (sex, grade, school environment and age) was prepared. The Preschoolers’ Body Scale was used [26] (Appendix A). This instrument has a male and a female version, and is composed of two scales with four real body figures each: the first showing four figures in frontal position, and the four in lateral position. Figures are presented in order from thinnest to most obese and all have the same height. The first figure represents a child with low weight (BMI-child = 13.13; BMI-girl = 13.03), the second one, a normal weight (BMI-child = 16; BMI-girl = 15.06), the third, an overweight child (BMI-child = 17.1; BMI-girl = 17.06), and the last one, an obese child (BMI-child = 21.03; BMI-girl = 21.25). Children were asked about what figure most suited them (perceived figure), and then, which figure they would like to have (desired figure). Thus, to determine body dissatisfaction, the value of the desired figure scale is subtracted from the value of the perceived figure scale. Authors reported a Fleiss’ Kappa (0.61) from the judgment of ten expert paediatricians and a test–retest correlation performed with children (ρfrontal = 0.40; ρlateral = 0.55).

### 2.3. Procedure

Hence, the database of public schools in the Autonomous Community of Extremadura (Spain) belonging to the Department of Education and Employment of the Regional Government of Extremadura was used to access the sample (available at: http://estadisticaeducativa.educarex.es/?centros/ensenanzas/&curso=17&ensenanza_centro=101200001 accessed on February 2022). Contact data were selected for those centres with the second stage of Early Childhood Education (3 to 6 years). An e-mail was then sent to the early childhood education teachers with information about the study and requesting their collaboration. The schools with an interest to participate were sent the informed consent form to be signed by the legal guardians. Then, a member of the research team went to every educational centre to collect data from the children. In the regular classroom, with both the researcher and the teacher present, the participants first filled in the socio-demographic questionnaire with the help of the teacher. The teacher indicated to the students whether they should mark rural or urban based on the characteristics of their school (previously agreed with the researcher based on whether the localities had more or less than 20,000 inhabitants. Those with less than 20,000 inhabitants were considered rural, as stated on the website of the Diputación de Cáceres (https://www.dip-caceres.es/, accessed on February 2022). Secondly, they were given The Body Scale for Preschoolers questionnaire. They were first given the first part of the questionnaire and they were tasked for approximately 5 min to think about and select the image that most resembled them. Then they were given the second part of the questionnaire and they were asked for 5 min to think about and select the image they would like to resemble (Appendix A). 

### 2.4. Statistical Analysis

Thus, to analyse if the collected data’s distribution met the assumption of normality, the Kolmogorov–Smirnov test was used, confirming the need to use nonparametric tests. The Mann–Whitney U test was used to analyze whether there were statistically significant differences between the figures perceived and the figures desired according to sex and centre location. Moreover, to analyze body dissatisfaction, perceived image and desired image data were treated independently for both the frontal and lateral scales and then the mean value obtained from the frontal scale and lateral scale was taken as a factor. Finally, Cronbach’s Alpha was used to calculate the reliability of each of the scales of the instrument.

## 3. Results

Table 1 shows the sample distribution according to sex, grade, and centre setting. The mean age of the participants was 4.42 years (SD = 0.82).

Table 2 shows the differences between the perceived (frontal and lateral) and desired (frontal and lateral) figures according to sex and centre location. Significant differences were found concerning the perceived figure according to sex, with girls obtaining higher values than boys. 

Table 3 shows descriptive data and differences in body dissatisfaction according to sex and centre location. Statistically significant differences were found according to sex, with girls obtaining higher body dissatisfaction than boys. No statistically significant differences were found in the scores obtained in the perceived figure, desired figure, and dissatisfaction as a function of the centre location.

Finally, the reliability of the results for each scale was 0.87 and 0.74 for the perceived and desired figure, respectively, all being satisfactory values above 0.70 according to Nunnally and Bernstein [27].

## 4. Discussion

This study arises from the need to understand body image perception in preschoolers to know its current state in Extremadura (Spain) schools in order to develop intervention programs. Differences in body image perception concerning sex have been widely studied in the literature, yielding different results over time. In this study, perceived image scores were higher than those according to the ideal image, contrasting with another study in the same population [28]; although in the literature, as in this research, there is a preference for more linear figures [29]. Children at this age usually have a negative perception of larger figures mainly due to sociocultural factors [14], more pronounced in females [12], as boys prefer muscular bodies, selecting those figures that represent an intermediate score even at a young age [30]. In this line, Canadian girls of these ages’ desire to be thinner are much higher than their male peers [13]. In contrast, children may select a slimmer or larger ideal figure than their own for reasons related to their body fat and musculature, or their desire for an adult form, which should not necessarily cause them to be worried about their current perceived size [31]. Other studies have reported no differences between sexes, such as Lowes and Tiggermann [32] with no differences in schoolchildren aged 3 to 5 years nor in the perceived or desired figure; or the one carried out by Pallan [33] finding no sex differences in self-rated body image and body dissatisfaction among South Asian preschoolers.

Little literature analyzes the environment as a possible factor affecting body image at an early age. In this study, no significant differences in body image were found between educational settings despite the rural children usually showing higher physical activity levels [34]. In this regard, Williams and his group [35] analyzed the children in rural Appalachia’s perceptions of their weight in comparison to their urban peers, finding no differences between groups. Gitau and associates [36] evaluated body image in African adolescent girls, reporting a greater preference for linear figures in girls living in urban areas. These results are in line with those obtained by Jackson [37], who discovered that, compared to rural females of the same age, a higher proportion of urban Egyptian girls between the ages of nine and eleven desired to be extremely skinny. Packard and Krogstrand [38] found that most of the study’s rural participants had at least one weight concern and had engaged in dieting and desired to be thinner.

The participants in our study showed higher values on body dissatisfaction, compared with another one conducted in a similar sample [39], although this could be explained by the non-inclusion of six-year-old schoolchildren, in whom sociocultural factors affect stronger body image [40]. Then, while some studies report body dissatisfaction in the majority of their participants [29], others report satisfaction levels above 50% [23]. This desire for a different body image is usually more linear [41], increases with age [42], and is more noticeable in girls [43]. Despite this, researchers such as Damiano [23] or Musher–Eizenman [44] have found that a high percentage of students, over 40%, indicated larger figures as ideal for them. In addition, this body dissatisfaction has been highly correlated with BMI, as students who are overweight or have obesity show higher levels of body dissatisfaction than those who are not [45]. Regarding the relationship between preschoolers’ body dissatisfaction and centre location, there is little analysis in the scientific literature. Leite [46] stated that school location, whether urban or rural, was unrelated to body dissatisfaction among Brazilian students. However, Ferguson and Cramer [47] found that children from rural backgrounds showed higher self-esteem levels than those residing in urban areas in the Jamaican population. By contrast, Welch [48] found that urban students between the ages of 9 and 11 had a higher ideal body image and were more content with their bodies.

This study has several limitations. Firstly, because of the convenience sampling used, findings must be interpreted with caution. Additionally, a limited number of sociodemographic factors is insufficient to deeply characterise the students considering the numerous environmental elements that impact children’s development. Information about social network use or relationships with parents or teachers is related to body image formation at an early age [49,50,51]. Another limitation was not objectively assessing participants’ body composition, so it would be interesting to consider objective methods for the body composition assessment. 

## 5. Conclusions

The current study focuses on analyzing the self-perceived body image of preschool children from the region of Extremadura, allowing them to evaluate body dissatisfaction levels in the early stages of development. Girls generally see themselves with a higher body mass index than boys and show greater body dissatisfaction. Centre location does not seem to be a variable to be considered in generating differences in body image, at least, in the current research.

Therefore, these results allow for the need to design and develop programs for body image in the early stages, considering sex differences.

## Figures and Tables

**Table 1:** Sample distribution (N = 304).

Variables	Categories	N	%
Sex	Boys	142	46.7
	Girls	162	53.3
Centre Location	Urban	168	55.3
	Rural	136	44.7
Grade	First	54	17.8
	Second	78	25.7
	Third	172	56.6

N: number; %: percentage.

**Table 2:** Descriptive values and differences in the scale according to sex and location of the centre.

		Sex			Centre Location		
Item	Total	Boys	Girls		Rural	Urban	
	M (SD)	M (SD)	M (SD)	*p*	M (SD)	M (SD)	*p*
1. Which child do you think looks most like you? (Frontal view)	2.43 (0.924)	2.23 (0.965)	2.60 (0.851)	<0.001 **	2.32 (0.892)	2.52 (0.941)	0.062
2. Which child do you think looks most like you? (Lateral view)	2.54 (0.866)	2.27 (0.939)	2.78 (0.722)	<0.001 **	2.49 (0.843)	2.59 (0.885)	0.244
3. Which child would you like to look like (Frontal view)?	1.68 (0.527)	1.65 (0.546)	1.70 (0.509)	0.368	1.62 (0.545)	1.73 (0.507)	0.049
4. Which child would you like to look like (Lateral view)?	1.71 (0.529)	1.67 (0.580)	1.74 (0.480)	0.152	1.65 (0.551)	1.76 (0.507)	0.061

M: mean; SD: Standard deviation; Note: The *p*-value is significant at ** *p* <0.01. Each score obtained in the dimensions is based on a scale (1–4).

**Table 3:** Descriptive data and differences in body dissatisfaction according to sex and centre locati.

		Sex			Centre Location		
Item	Total	Boys	Girls		Rural	Urban	
	M (SD)	M (SD)	M (SD)	*p*	M (SD)	M (SD)	*p*
Body dissatisfaction (Frontal view)	−0.75 (0.876)	−0.57 (0.885)	−0.90 (0.843)	<0.001 **	−0.69 (0.871)	−0.79 (0.881)	0.491
Body dissatisfaction (Lateral view)	−0.83 (0.874)	−0.60 (0.906)	−1.03 (0.795)	<0.003 **	−0.83 (0.896)	−0.83 (0.859)	0.993
Body dissatisfaction (Mean value)	−0.79 (0.791)	−0.59 (0.782)	−0.96 (0.759)	<0.001 **	−0.76 (0.783)	−0.81 (0.799)	0.641

M: mean; SD: Standard deviation; Note: The *p*-value is significant at ** *p* <0.01. Each score obtained in the dimensions is based on a scale (1–4).

## Data Availability

The datasets are available through the corresponding author on reasonable request.

## Appendix A

### Appendix A.1. Escala Corporal para Preescolares -Versión niño

Datos Sociodemográficos

Edad:
Curso:
Colegio:
  □ Rural
  □ Urbano

¿Qué niño se parece más a ti?

¿A qué niño te gustaría parecerte?

### Appendix A.2. Escala Corporal para Preescolares -Versión niña

Datos Sociodemográficos

Edad:
Curso:
Colegio:
  □ Rural
  □ Urbano

¿Qué niña se parece más a ti?

¿A qué niña te gustaría parecerte?
